# Synthesis and Antiplasmodial Activity of Betulinic Acid and Ursolic Acid Analogues

**DOI:** 10.3390/molecules171012003

**Published:** 2012-10-12

**Authors:** Adrine M. Innocente, Gloria N. S. Silva, Laura Nogueira Cruz, Miriam S. Moraes, Myna Nakabashi, Pascal Sonnet, Grace Gosmann, Célia R. S. Garcia, Simone C. B. Gnoatto

**Affiliations:** 1 Laboratório de Fitoquímica e Síntese Orgânica, Faculdade de Farmácia, Programa de Pós-Graduação em Ciências Farmacêuticas, Universidade Federal do Rio Grande do Sul. Av. Ipiranga, 2752, Porto Alegre 90610-000, RS, Brazil; Email: a_innocente@yahoo.com.br (A.M.I.); glorianarjara@yahoo.com.br (G.N.S.S.); grace.gosmann@ufrgs.br (G.G.); 2 Laboratório de Biologia Celular e Molecular de *Plasmodium*, Departamento de Fisiologia, Instituto de Biociências, Universidade de São Paulo, USP. Rua do Matão, travessa 14, 321, São Paulo 05508-900, SP, Brazil; Email: laurancruz@gmail.com (L.N.C.); miradun@yahoo.com.br (M.S.M.); mnakab@ib.usp.br (M.N.); cgarcia@usp.br (C.R.S.G.); 3 Laboratoire des Glucides, FRE CNRS 3517, UFR de Pharmacie Université de Picardie Jules Verne1, Rue des Louvels, 80037 Amiens cedex 1, France

**Keywords:** triterpenes, betulinic acid, ursolic acid, malaria, *P. falciparum*, cytotoxicity, calcium

## Abstract

More than 40% of the World population is at risk of contracting malaria, which affects primarily poor populations in tropical and subtropical areas. Antimalarial pharmacotherapy has utilised plant-derived products such as quinine and artemisinin as well as their derivatives. However, worldwide use of these antimalarials has caused the spread of resistant parasites, resulting in increased malaria morbidity and mortality. Considering that the literature has demonstrated the antimalarial potential of triterpenes, specially betulinic acid (**1**) and ursolic acid (**2**), this study investigated the antimalarial activity against *P. falciparum *chloroquine-sensitive 3D7 strain of some new derivatives of **1** and **2** with modifications at C-3 and C-28. The antiplasmodial study employed flow cytometry and spectrofluorimetric analyses using YOYO-1, dihydroethidium and Fluo4/AM for staining. Among the six analogues obtained, compounds **1c** and **2c** showed excellent activity (IC_50_ = 220 and 175 nM, respectively) while **1a** and **b** demonstrated good activity (IC_50_ = 4 and 5 μM, respectively). After cytotoxicity evaluation against HEK293T cells, **1a** was not toxic, while **1c** and **2c** showed IC_50 _of 4 μM and a selectivity index (SI) value of 18 and 23, respectively. Moreover, compound **2c**, which presents the best antiplasmodial activity, is involved in the calcium-regulated pathway(s).

## 1. Introduction

Malaria is responsible for some 216 million clinical cases and 655,000 deaths annually, especially among children and pregnant women [1]. Due to the spread of resistant parasites, the development of new medicines is imperative for the control and eradication of malaria [2]. 

Among the first antimalarial substances, the cinchona alkaloids (such as quinine and quinidine) were shown to arrest the development of malignant tertian malaria parasites. Subsequently, the sesquiterpene lactone artemisinin was obtained from *Artemisia annua*, and became very important for the control of malarial infection, resulting in the development of more potent derivatives, such as artemether and artesunate [3]. Thus the partnership between medicinal and natural products chemistry is an important strategy in innovative drug discovery [4,5]. In this sense, the chemical modification of natural products having antimalarial activities may be one promising method for developing more effective and less toxic antimalarial drugs.

**Figure 1:**
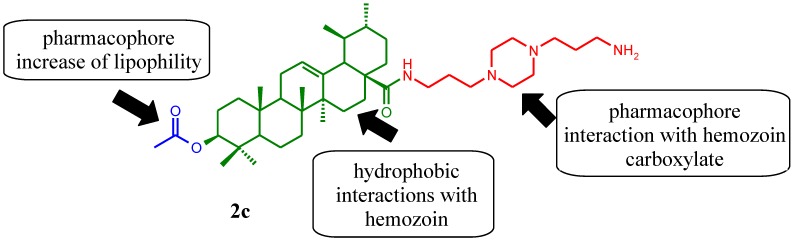
Strategy used to obtain *N*-{3-[4-(3-aminopropyl)piperazinyl]propyl}-3-*O*-acetylursolamide derivatives as antimalarial agents.

Many studies regarding triterpenes and their derivatives possessing antimalarial activity which highlight two triterpenes, betulinic and ursolic acids, are reported [6,7,8,9,10]. For example, Ziegler and co-workers described the anti-*Plasmodium* activity on the 3D7 strain of betulinic acid and its analogues and reported that treatment with these compounds resulted in modified erythrocyte membranes. While betulinic acid was active, with an IC_50 _value of 13.9 µM, the most active analogue was methyl betulinate (IC_50_ 7 µM) [7]. However, in a murine malarial model of *P. berghei* betulinic acid was ineffective at 250 mg/kg/day [6]. We developed some piperazinyl derivatives of ursolic acid obtaining three antimalarial compounds potent against the *P. falciparum* chloroquine-sensitive Thai strain, including compound **2c** possessing an IC_50_ value of 167 nM [10] (Figure 1). Moreover, we could demonstrate the importance of the triterpene skeleton bearing an acetyl group at C-3 and the piperazine moiety was also identified as a pharmacophore.

These derivatives were designed based on studies reporting that piperazine derivatives have antimalarial action [11,12]. For example, a series of bisacridine derivatives coupled with bisaminopropylpiperazine were found to have significant antimalarial effects when tested *in vitro* for antimalarial activity against the *P. falciparum* chloroquine-resistant FcB1R strain [11].

Until the moment little is known about mechanism of action for triterpenes, with reports for hematin and modification at erythrocyte membrane actions. [7,10]. Because Ca^2+^ homeostasis and signalling are fundamental for parasite survival [13,14,15], the mechanism involved in calcium homeostasis within malaria parasites has been investigated as a potential target against malaria [16].

In continuation of our research, the present study investigated new piperazinyl derivatives at C-28 of acetyl betulinic acid (compounds **1a**–**c**) comparing them with those of the ursolic acid (compounds **2a**–**c**) as prototypes for new antimalarial compounds against the *P. falciparum *chloroquine-sensitive 3D7 strain. Further the cytotoxicity *in vitro* was estimated and a novel antimalarial mechanism of action for triterpenes was also revealed. 

## 2. Results and Discussion

### 2.1. Chemical Synthesis

**Scheme 1:**
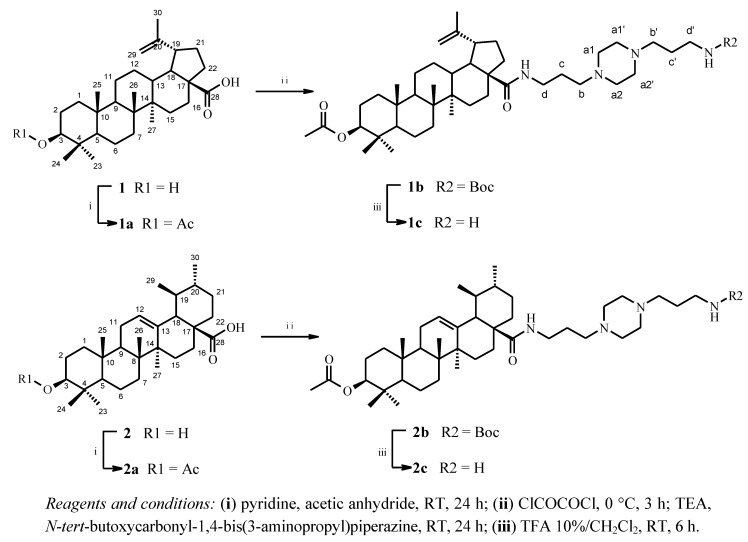
Synthesis of *N*-{3-[4-(3-aminopropyl)piperazinyl]propyl}-3-*O*-acetyl betulinamides **1a**–**c** and *N*-{3-[4-(3-aminopropyl)piperazinyl]propyl}-3-*O*-acetylursolamides **2a**–**c**.

After being isolated from natural sources, the importance of the C-3 hydroxyl group of betulinic and ursolic acids **1** and **2** was ascertained through usual acetylation (Scheme 1). The acetylated compounds numbered **1a** and **2a** were obtained in 98% yield. The coupling reaction of **1a** and **2a **with *N-tert*-butoxycarbonyl-1,4-bis(3-aminopropyl)piperazine occurred following activation of the carboxylic acid at C-17 with oxalyl chloride as previously described [10]. The corresponding derivatives **1b** and **2b** were then isolated in 80% yield. Treatment with 10% trifluoroacetic acid/dichloromethane removed the *tert*-butoxycarbonyl (Boc) to provide compounds **1c** and **2c** in 70% yield (Scheme 1). All compounds were fully characterised by spectroscopic data. 

### 2.2. Antiplasmodial Assay

**Figure 2:**
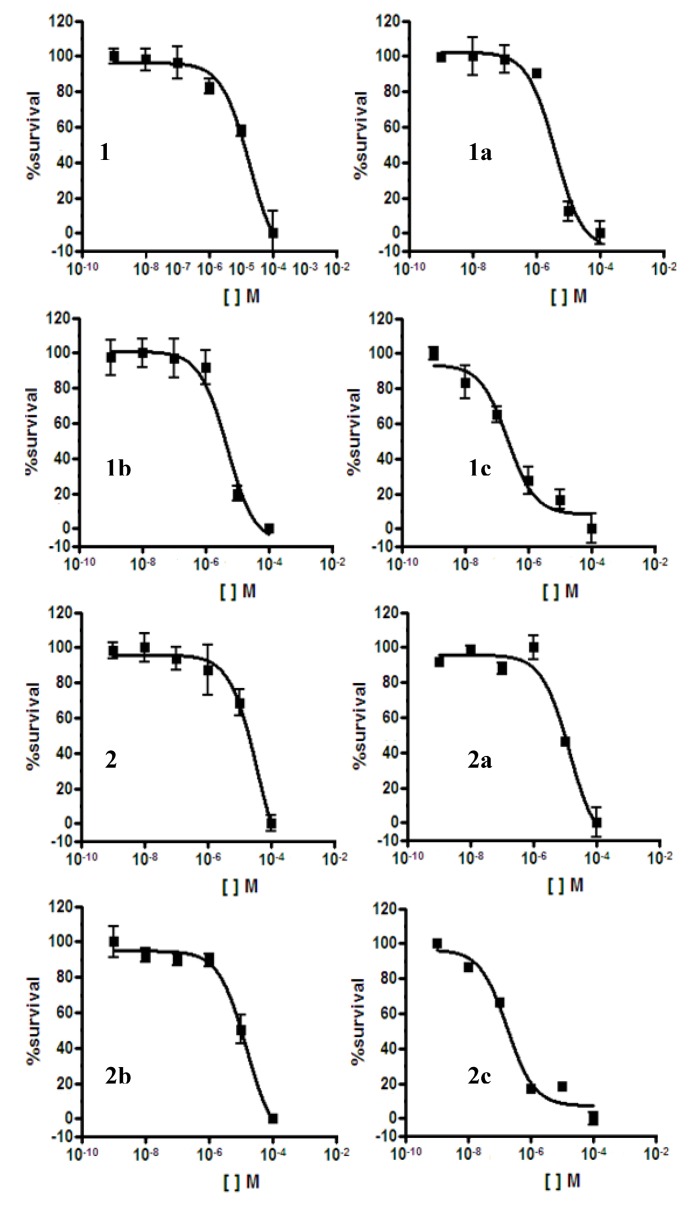
Drug concentration response curves of betulinic and ursolic acid acetylated derivatives **1a** and **2a**, coupled **1b** and **2b** and deprotected piperazinyl derivatives **1c** and **2c** against the chloroquine-sensitive *P. falciparum *strain 3D7 strain. The results are representative of three independent experiments (n = 3).

All compounds were evaluated *in vitro* against the *P. falciparum *chloroquine-sensitive 3D7 strain in comparison to chloroquine which IC_50_ value was 29 nM. Compound **1** showed moderate activity (IC_50_ 18 µM) and **2** was inactive (IC_50_ 36 µM) (Figure 2, Table 1) which results are in agreement with those of the literature [6,17,18].

Acetylated derivatives **1a** and **2a** showed better antimalarial activity than their aglycones (IC_50_ values of 4 and 14 µM, respectively) (Figure 2, Table 1). The highest values were obtained for the piperazinyl derivatives **1c** (IC_50_ 220 nM) and **2c** (IC_50_ 175 nM). Protected compounds **1b** and **2b** presented IC_50_ values of 5 and 15 µM, respectively, demonstrating a lower capacity to reduce parasitemia in comparison to unprotected compounds. As shown in Figure 2, the piperazine group contributed to a significant improvement in activity as also reported [19].

### 2.3. Cytotoxicity Assay

The active derivatives **1a**–**c** and **2c** were evaluated for their cytotoxicity in human embryonic kidney cells (HEK293T) by monitoring the number of living cells at the same concentrations used to evaluate the antiplasmodial activity (100, 10, 1, 0.1, 0.01 and 0.001 µM). Many research groups have tried to find cytotoxicity activity from triterpenoids, including betulinic acid and their derivatives. Thibeault *et al.* presented cytotoxicity results of lupane-type triterpenoid glyceryl esters. Betulinic acid showed values of 18 to 57 µM in several cell lines [20].

**Table 1:** Antiplasmodial and cytotoxic activity of compounds **1**, **1a**–**c** and **2**, **2a**–**c**. The results are representative of three independent experiments (n = 3).

Compounds	IC_50_ (µM) *P. falciparum *3D7	IC_50_ (µM) HEK293T		Selectivity Index (SI) 48 h
		24 h	48 h	
1	18	-	-	-
1a	4	>100	>100	-
1b	5	4	4	0.8
1c	0.220	4	4	18
2	36	-	-	-
2a	14	-	-	-
2b	15	-	-	-
2c	0.175	4	4	23

On the other hand, the cytotoxicity of heterocyclic triteprenoids derived from betulin and betulinic acid was tested against seven tumor and two normal fibroblast cell lines showing activity values from 1.0 to 55.5 µmol/L [21]. The results displayed in Table 1 show that **1a** was not cytotoxic at the tested concentrations, while the piperazinyl derivatives **1b**, **c** and **2c** showed IC_50_ values of 4 µM. Moreover, the most active compounds **1c** and **2c** presented SI values of 18 and 23, respectively, being considered non-toxic.

### 2.4. Infected Erythrocytes: Loading with the Calcium Indicator Fluo4/AM

Like most eukaryotic cells, malaria parasites have internal Ca^2^^+ ^pools responsible for regulation of signalling processes during invasion, maturation and division that can be mobilised upon agonist stimulation in the plasma membrane [22]. The effect of compound **2c **through modulation of one or more calcium pathways in *P. falciparum *(3D7) was demonstrated using the fluorescent calcium probe Fluo4/AM. This molecule was chosen because it has the highest selectivity index (23, Table 1). 

**Figure 3:**
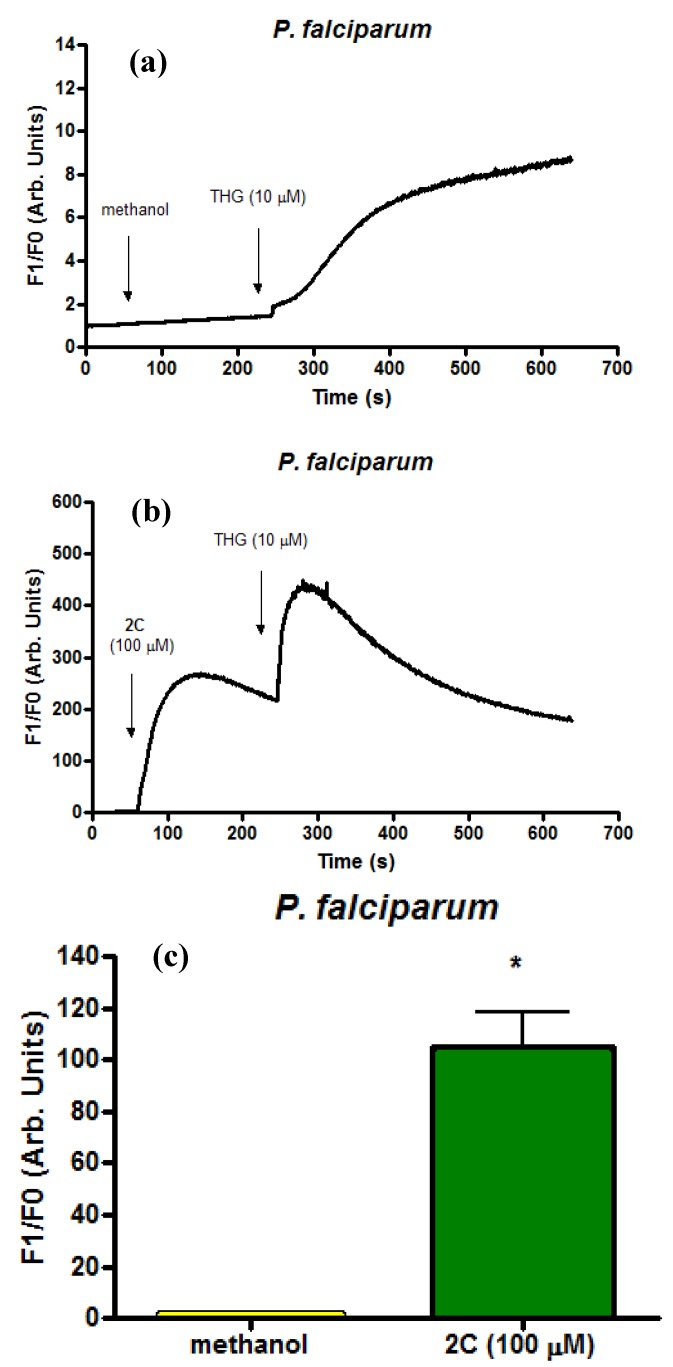
Modulation of the *Plasmodium *calcium pathway(s) involved in the mechanism of action of compound **2c**. Representative traces of the change in Fluo4/AM) fluorescence over time upon addition of solvent (**a**) or **2c** (100 µM) (**b**) in *P. falciparum* followed by THG (10 µM) addition. (**c**) Bar graph analysis of Ca^2+^ concentrations in Fluo4/AM-labelled *P. falciparum *isolated parasites (10^8^ cells mL^−1^) after addition of **2c** (100 µM). Data presented (104.9 a.u. ± 13.65, n = 9, *P *= 0.0004) were in *P. falciparum.* *P* values (*) were calculated by comparison with methanol (0.05%) (1.46 a.u. ± 0.057, n = 4). Bars represent the means and SEM values. The fluorescence was measured continuously (acquisition every 0.5 s). The results are representative of three independent experiments performed in triplicate.

The typical rise in cytosolic calcium in isolated parasites after addition of compound **2c** (100 µM) and thapsigargin (THG) (10 µM) is presented in Figure 3a–c. Because THG has been known to modulate Ca^2+^ via inhibition of sarco-endoplasmatic reticulum Ca^2+^ ATPase (SERCA) [23], it was used as the control in this experiment. The control experiment (with methanol addition) showed clearly that the solvent did not elicit calcium increase within the parasites (Figure 3a). On the contrary, when THG was added after compound **2c**, the calcium increase was greater than the conventional calcium mobilisation observed with the control one (Figure 3b). These results indicated that **2c** can modulate parasite calcium homeostasis.

## 3. Experimental

### 3.1. General

Infrared spectra (IR) were obtained using a PerkinElmer FT-IR System Spectrum BX instrument. ^1^H and ^13^C-NMR spectra were recorded on Varian Inova 300 and Varian VNMRS 300 spectrometers using TMS as internal standard. Chemical shifts are reported in parts per million (δ). An UltrOTOF (BrukerDaltonics) mass spectrometer was used to obtain HR-EI-MS spectra. Melting points were determined using a Koffler instrument. All reactions were monitored by thin-layer chromatography on Merck silica plates GF using different solvent systems. Column chromatography was carried out on silica gel GF (Merck, 60–230 mesh) using gradient mixtures (CH_2_Cl_2_/methanol). All reagents were purchased as analytical grade. All solvents were dried and distilled prior use. Dichloromethane was dried over CaCl_2_ for 24 h then was submitted to reflux. 

### 3.2. Plant Material and Isolation of Triterpenes

The betulinic acid was obtained from *Platanus acerifolia*. A voucher specimen (ICN 182537) is on deposit in the Botany Department Herbarium of Universidade Federal do Rio Grande do Sul, Porto Alegre, Brazil. The peel of *Malus domestica *was a byproduct from the juice industry company Tecnovin S.A., Vacaria, RS, Brazil, and was used to obtain the ursolic acid. Both powdered dried materials were separately subjected to reflux with ethanol 60% (250 mL, 2 h). Then, after removing the ethanol under vacuum, each residual aqueous phase was separately extracted with ethyl acetate, and the organic phase was evaporated to dryness to obtain a residue that was submitted to recrystallisation. The betulinic acid was recrystallized from methanol, while ursolic acid was recrystallized using acetonitrile. *Betulinic acid *(**1**). Yield 1.5%. Its structure was confirmed by spectroscopic methods which were in agreement with literature data [6,7]. *Ursolic acid *(**2**). Yield 4%. Its structure was confirmed by spectroscopic methods and literature data [10]. 

### 3.3. Chemical Synthesis

The ursolic acid derivatives were prepared using a semisynthetic route developed by Gnoatto and co-workers which details of the chemistry and structural elucidation were described previously [10]. Briefly, the acetylated ursolic acid **2a** was submitted to a coupling reaction with *N*-Boc-bisaminopropylpiperazine in basic conditions to obtain **2b**, and then the deprotected analogue **2c** was obtained by removing the *N*-Boc-amino group. All structural assignments were compared with the published data [10]. The betulinic acid derivatives were obtained using an analogous route. 

*3-O-Acetylbetulinic acid *(**1a**). Yield 98%. It was prepared from the corresponding aglycone **1** by usual acetylation [10]. 

*N-{3-[4-(3-Aminopropyl)piperazinopropyl]terbutylcarbamate}-3-acetylbetulinamide* (**1b**). Oxalyl chloride (0.15 mL, 1.7 mmol) was added to **1a** (285 mg, 0.57 mmol) dissolved in CH_2_Cl_2_ (30 mL) at 0 °C, and the resulting solution was stirred under N_2_ for 3 h. Subsequently, triethylamine (0.5 mL, 3.4 mmol) and then *tert*-butyl 3-[4-(3-aminopropyl)piperazinylpropyl]carbamate (510 mg, 1.7 mmol) were added to the reaction, which was then stirred for 24 h at room temperature. Next, water was added and the resulting product was extracted with CH_2_Cl_2_ (3 × 30 mL); the organic layers were combined, dried over Na_2_SO_4_ and evaporated under vacuum to afford **1b** as a white solid. Yield: 80%; m.p. 129 °C; IR (ATR cm^−1^): 3406 (NH); 2943 (C-H); 1777 (C=O acetyl); 1687 (C=O amide); 1555 (C=C); 1425 (C-O); 1380 (C-H); 1259 (C-O-C); 1176 (C-N). ^1^H-NMR (CDCl_3_, δ): 0.84 (s, 3H, H_26_); 0.85 (s, 3H, H_27_); 0.87 (s, 3H, H_25_); 0.95 (s, 3H, H_23_); 0.97 (s, 3H, H_24_); 1.29 (d, 4H, H_11_, H_12_); 1.38 (m, 3H, H_6_, H_9_); 1.45 (s, 9H, H_3_C-Boc); 1.55 (t, 4H, H_15_, H_7_); 1.65 (m, 6H, H_13_, 2 × CH_2_, H_5_); 1.69 (s, 3H H_30_); 1.76 (t, 4H, H_16_, H_1_) 1.97 (t, 3H, H_18_, H_2_); 2.04 (s, 3H, H_3_C-COO-); 2.47 (m, 15H, H_1_, 7 × CH_2_); 3.19 (m, 2H, CH_2_); 3.32 (m, 4H, 2 × CH_2_); 4.48 (dd, 1H, H_3_); 4.59 (brs,1H, H_29a_); 4.75 (brs, 1H, H_29b_); 5.36 (1H, NH); 6.86 (1H, NH). ^13^C-NMR (CDCl_3_, δ): 14.55; 16.14; 16.22; 16.43; 18.17; 19.46; 20.94; 20.20; 23.68; 21.22 (H_3_C-COO-); 25.57; 26.42; 28.41 (H_3_C-Boc); 29.41; 30.91; 33.84; 34.93; 37.12; 37.58; 37.77; 38.39; 38.46; 39.55; 39.87; 40.76; 42.42; 46.68; 50.20; 50.57; 53.10; 53.45; 55.48 (4C); 56.73; 57.85; 75.80; 78.79 (Cq-Boc); 80.93; 109.23; 151.04; 156.0 (CO-Boc); 170.90 (H_3_C-COO-); 176.14 (C-28). HRMS (ESI-MS, *m/z*); [M+H]^+^: Calcd. for C_47_H_80_N_4_O_5_: 781.1619; Found: 781.6175. 

*N-{3-[4-(3-aminopropyl)piperazinyl]propyl}-3-acetylbetulinamide* (**1c**). The *N*-Boc-amino protecting group of compound **1b** was removed using trifluoroacetic acid 10%/dichloromethane mixture according to the literature [10], resulting in the deprotected analogue **1c**. Yield 70%; m.p. 120 °C. Its spectroscopic data were similar to those of **1b**, excepting for the absence of the *N*-Boc-amino signals. HRMS (ESI-MS, *m/z*), [M+H]^+^: Calcd. for C_40_H_72_N_4_O_3_: 681.0461; Found: 681.5712. 

### 3.4. Antiplasmodial Assay

The chloroquine-sensitive 3D7 strain of *P. falciparum* was grown in RPMI (Gibco/Life Technologies, Grand Island, NY, USA) containing 37.5 mM HEPES, supplemented with 7 mM D-glucose, 6 mM NaOH, 25 mL gentamicin sulphate, 2 mM L-glutamine and 10% human serum albumin. The strains were maintained in human erythrocytes in a gas mixture containing 5% O_2_, 5% CO_2_ and 90% N_2_ [24]. The culture was synchronised by 10% sorbitol treatment [25]. The antimalarial activity was measured using the microdilution technique *in vitro*. Red cells with parasitemia between 1–2% and 2% hematocrit were incubated with the compounds **1**, **1a**–**c** and **2**, **2a**–**c** for 48 h. Samples were dissolved in water, dimethylsulfoxide (DMSO) or methanol and serially diluted with the culture medium. To evaluate parasitemia, Giemsa staining and flow cytometric analysis were used. The concentration of the drug required to inhibit parasite growth was determined by comparing the fluorescence incorporated into the treated and untreated cultures (control). The concentration causing 50% inhibition (IC_50_) was determined from the drug concentration response curve. Methanol and DMSO were used at the maximum concentration of 0.1% and did not inhibit parasite growth.

### 3.5. Cytotoxicity Assay

Human embryonic kidney (HEK293T) cells were cultured in surface area 75 cm^2^ (Greiner Bio-One, Frickenhausen, Germany) vented tissue culture flasks at 37 °C in a humidified atmosphere containing 5% CO_2_ in Dulbecco’s modified essential medium (Gibco/Life Technologies, Grand Island, NY, USA) supplemented with 10% (v/v) foetal bovine serum, 100 U/mL penicillin and 100 µg/mL streptomycin. After 24 h, 50 µl of each drug was added with 0.5% DMSO or methanol to a single well. The cells were incubated with the test drugs for an additional 24 and 48 h and then harvested with trypsin, gently pelleted by centrifugation and re-suspended in PBS. The cells were stained with dihydroethidium solution (10 mg/mL in phosphate buffered saline), gently vortexed and incubated for 40 min at 37 °C in the dark. Ten thousand gated events were acquired for each sample (FACSCalibur BD and CellQuest software was used for analysis).

### 3.6. Infected Erythrocytes: Loading with the Calcium Indicator Fluo4/AM

Erythrocytes (RBCs) infected by *P. falciparum* (3D7) at the trophozoite stage were lysed with 10 mg/mL saponin (in phosphate-buffered saline) in the presence of 20 µg/mL protease inhibitors (pepstatin A, leupeptin, antipain and chymostatin) and benzamidine (0.5 mmol/L). Erythrocyte membranes were then removed through centrifugation (8700 rpm for 10 min at 4 °C). The parasites were washed three times in buffer M (116 mM NaCl, 5.4 mM KCl, 0.8 mM MgSO_4_, 5.5 mM *D*-glucose, 50 mM MOPS, pH 7.3) with CaCl_2_ (2 mM). The parasites were then re-suspended in the same buffer with Fluo4-AM (5 μM) probenecid (1.8 mM), an inhibitor of organic anion transport (to prevent dye sequestration and release), 20 mg/mL protease inhibitors (pepstatin A, leupeptin, antipain and chymostatin) and benzamidine (0.5 mmol/L) for 50 min at 37 °C. The parasite suspension was then washed three times with buffer M. Spectrofluorimetric measurements were performed in a Shimadzu RF-5301 PC instrument at 37 °C with isolated *P. falciparum* (3D7) parasites (10^8^ cells mL^−1^) incubated with MOPS buffer in a 1 mL cuvette. Compound **2c** (100 μM) and THG (10 μM) were added during time course experiments, and excitation/emission wavelengths were adjusted to 505/530 nm for Fluo4-AM. Methanol (0.05%) was used as a control. Addition of the detergent digitonin induced the maximum increase in calcium that was abolished by addition of the calcium chelator EGTA.

### 3.7. Statistical Analysis

Student’s test was chosen for comparisons between two groups, whereas repeated ANOVA measures were used for comparisons among more than two groups. The results are expressed as the mean ± SEM of at least three individual experiments. *P *< 0.05 was considered a statistically significant difference. GraphPad Prism software (San Diego, CA) was used for all statistical tests.

## 4. Conclusions

In this study, we synthesized new piperazinyl derivatives of betulinic and ursolic acids considering the previous active designed compounds. The best compounds, **1c **and **2c**, showed IC_50_ values in the range of 175 to 220 nM against the *P. falciparum* chloroquine-sensitive 3D7 strain, and they are considered non-toxic (SI > 10 μM). The observed changes in calcium homeostasis demonstrated that ursolic acid derivative **2c** led to an increase in intracellular ion concentrations in *P. falciparum. *In conclusion, compound **2c** is a new potent antimalarial prototype that disrupts *Plasmodium *calcium homeostasis. 
